# The role of stress cardiac MRI imaging in patients with complete left bundle branch block

**DOI:** 10.1186/1532-429X-14-S1-P150

**Published:** 2012-02-01

**Authors:** Balaji Rao, Yucheng Chen, Raymond Kwong

**Affiliations:** 1 Non Invasive Cardiovascular Imaging, Brigham and Women's Hospital, Boston, MA, USA; 2 Cardiology, West China Hospital, Chengdu, China

## Background

Left bundle branch block (LBBB) is a condition associated with increased mortality and poor prognosis, depending on the underlying etiology. Identification of the etiology and appropriate risk stratification of patients with LBBB is a challenge, despite the availability of various testing modalities. Nuclear exercise stress testing can show artificial perfusion defects in the interventricular septum. The artifacts are reduced but not completely excluded by use of vasodilator stress agents. In this study, we retrospectively reviewed the stress cardiac magnetic resonance (CMR) findings in patients with LBBB.

## Methods

We retrospectively reviewed 49 consecutive patients with complete LBBB who underwent stress CMR. Of these 49 patients, 23 also underwent concurrent coronary angiography, 20 underwent nuclear stress test and 8 underwent all the three tests. The reference standard for flow limiting coronary artery disease (CAD) was coronary artery stenosis greater than 50% on coronary angiogram.

## Results

Stress CMR correctly identified all patients (n=13) with more than 50% coronary artery stenosis and all patients (n=7) without significant coronary artery stenosis.

Overall, the sensitivity, specificity, positive predictive value and negative predictive value of CMR in diagnosing flow limiting CAD were 100%, 70%, 81% and 100% respectively.

In patients who underwent both stress CMR and nuclear stress test (n=20), stress CMR showed late gadolinium enhancement (LGE) in the coronary artery distribution in thirteen patients (n=13 of 20), consistent with fibrosis due to myocardial infarction. Where as nuclear stress test showed fixed defect in a coronary artery distribution in only six patients (n=6 of 20).

Stress CMR also demonstrated other etiologies like hypertrophic cardiomyopathy, cardiac iron overload and idiopathic dilated cardiomyopathy.

## Conclusions

Stress CMR imaging has high sensitivity and negative predictive value in the identification of CAD, in patients with complete LBBB. It can also assess infiltrative cardiomyopathies. Stress CMR appears to provide a better comprehensive assessment than nuclear scintigraphy in patients with LBBB.

## Funding

None.

